# USP5 regulates ferroptosis in colorectal cancer by targeting the YBX3/SLC7A11 axis through lysosomal degradation

**DOI:** 10.1038/s41419-025-08146-2

**Published:** 2025-11-10

**Authors:** Haowen Qiu, Yi Liu, Haimeng Zhou, Lingjuan Hu, Wei Qi, Honglu Ma, Yaoyi Liu, Le Li, Nanyang Yang, Meiqin Huang, Runlei Du, Lijuan Meng, Feng Shi, Baiqi Wang, Li Yu, Xiaodong Zhang, Guoqing Li

**Affiliations:** 1 National Health Commission Key Laboratory of Birth Defect Research and Prevention & MOE Key Lab of Rare Pediatric Diseases, Institute of Cell Biology and Genetics, School of Basic Medical Sciences, Hengyang Medical School, University of South China, Hengyang, Hunan China; 2 College of Life Sciences & Renmin Hospital, Wuhan University, Wuhan, Hubei China; 3 The Second Affiliated Hospital, Hengyang Medical School, University of South China, Hengyang, Hunan China; 4 Key Laboratory of Research on Clinical Molecular Diagnosis for High Incidence Diseases in Western Guangxi of Guangxi Higher Education Institutions, Affiliated Hospital of Youjiang Medical University for Nationalities, Baise, Guangxi China

**Keywords:** Oncogenes, Cell death

## Abstract

Colorectal cancer (CRC) is the third most common cancer worldwide and a significant public health threat. Ferroptosis, an iron-dependent form of regulated cell death, has emerged as a promising therapeutic target in CRC treatment. Despite its significant clinical potential, the precise regulatory mechanisms underlying ferroptosis, particularly its role in ferroptosis within CRC, remain to be fully elucidated. Previous studies, including our own work, have revealed that various deubiquitinases (DUBs) are involved in regulating cellular processes; however, the specific mechanisms by which these enzymes contribute to ferroptosis in CRC remain unclear. In this study, we identify USP5 as a key regulator of ferroptosis in CRC. Traditionally recognized as a deubiquitinase, USP5 modulates cellular physiological activities through deubiquitination. However, our findings show that USP5, distinct from its conventional deubiquitination function, suppresses ferroptosis by promoting the lysosomal degradation of YBX3 (Y-box binding protein 3). Under normal conditions, YBX3 promotes the degradation of SLC7A11 (solute carrier family 7 member 11). However, USP5 facilitates the degradation of YBX3, leading to the stabilization of SLC7A11 and thereby promoting CRC cell survival and tumor progression. In patient-derived organoid and xenograft models, USP5 knockout significantly increased the sensitivity of cancer cells to ferroptosis and inhibited tumor growth. Moreover, additional knockout of YBX3 restored the stability of SLC7A11, highlighting the complex regulatory network between USP5, YBX3, and SLC7A11. Systematic functional assays and mechanistic studies further confirmed that the USP5/YBX3/SLC7A11 axis is a central pathway for ferroptosis resistance in CRC. These findings provide novel insights into therapeutic strategies for CRC, especially ferroptosis-based treatments.

Colorectal cancer (CRC) is a commonly diagnosed malignancy worldwide and ranks as the second leading cause of cancer-related deaths globally, and poses a major public health threat [1–3]. According to the latest statistics from 2024, China reports around 510,000 new CRC cases and approximately 240,000 deaths each year [4]. In the United States, there are an estimated 152,810 new CRC cases in 2024, resulting in about 53,010 deaths [1]. Current treatment options for CRC include chemotherapy, radiotherapy, immunotherapy, and surgery [5–7]. However, these therapeutic approaches are often hampered by high rates of recurrence, intrinsic or acquired drug resistance, and significant adverse effects, underscoring the pressing need for more effective and targeted treatment strategies [8, 9].

Ferroptosis is a regulated form of cell death characterized by iron-dependent lipid peroxidation, leading to oxidative damage of cellular membranes [10]. This process is initiated through intracellular iron-catalyzed lipid peroxidation and is morphologically defined by mitochondrial shrinkage and the reduction or loss of cristae structure [11–14]. Unlike apoptosis and necrosis, ferroptosis is orchestrated by cellular metabolic activity, redox equilibrium, and iron homeostasis [15, 16]. Central regulators of this pathway include glutathione peroxidase 4 (GPX4) and the cystine/glutamate antiporter system Xc⁻, particularly its subunit SLC7A11 [17, 18]. The role of ferroptosis in cancer biology extends beyond its intrinsic cytotoxicity toward malignant cells [19, 20]. While the induction of ferroptosis may suppress tumor progression, paradoxically, it may also facilitate cancer development under specific microenvironmental conditions [21, 22]. For instance, DHPO, an allosteric covalent inhibitor of USP7, has been shown to inhibit gastric cancer proliferation and metastasis both in vitro and in vivo by downregulating stearoyl-CoA desaturase (SCD) and promoting ferroptosis [23]. Conversely, circPIAS1 has been found to suppress ferroptosis by competitively binding to miR-455-3p, thereby upregulating Nuclear Protein 1 (NUPR1), which facilitates the growth and metastatic potential of hepatocellular carcinoma (HCC) [24]. Emerging evidence suggests that CRC cells often exhibit elevated intracellular iron levels and dysregulated lipid metabolism, rendering them particularly susceptible to ferroptosis induction [25, 26]. This unique vulnerability highlights ferroptosis as a promising therapeutic target, especially in the context of drug-resistant CRC [27, 28].

Ubiquitination is a critical post-translational modification that regulates numerous cellular functions, particularly protein degradation and stabilization [13, 29]. Ubiquitin-specific peptidase 5 (USP5), a member of the deubiquitinating enzyme (DUB) family, primarily functions to deubiquitinate substrate proteins, thereby modulating essential biological processes such as proteostasis [30–33]. Recent studies have demonstrated that USP5 expression is significantly upregulated in breast cancer cells, where it promotes cell proliferation and migration, potentially through the stabilization of HIF-2α [34]. In non-small cell lung cancer, USP5 has been implicated in enhancing cellular proliferation and resistance to apoptosis by stabilizing PD-L1 [35]. Similarly, in bladder cancer, elevated USP5 expression facilitates tumor progression via stabilization of c-Jun [36]. Beyond its canonical role in ubiquitin processing, USP5 is also involved in cellular stress responses independent of its deubiquitinase activity [30, 37, 38]. Moreover, it plays a pivotal role in DNA damage repair and the maintenance of genomic stability, indirectly contributing to tumor cell survival and chemoresistance [39–41].

YBX3, also known as CSDA, is a multifunctional nucleic acid-binding protein that belongs to the Y-box-binding protein (YBX) family. It plays a critical role in a variety of essential cellular processes, primarily including the regulation of gene expression, RNA metabolism, cell proliferation, differentiation, and responses to cellular stress [42, 43]. In bladder cancer (BC), lncRNA BLACAT3 facilitates the nucleocytoplasmic shuttling of YBX3, enhancing its binding to the promoter of the target gene *NCF2* and thereby promoting angiogenesis and hematogenous metastasis [44]. In prostate cancer (PCa), the m6A reader PROPER mediates the formation of a complex between YBX3 and YTHDF2, which promotes the degradation of DUSP1, consequently driving tumor metastasis [45]. Furthermore, in gastric cancer (GC), YBX3 regulates Hepatocyte Growth Factor (HGF)-mediated cellular proliferation and metastasis [46]. Notably, the related family member YBX1 has been shown to modulate ferroptosis in endometrial cancer cells by regulating SLC7A11, highlighting the significant implication for investigating the potential role of YBX3 in ferroptosis regulation [47].

In CRC, iron metabolism is frequently dysregulated [48]. Inducing ferroptosis can enhance cancer cell death, whereas inhibiting ferroptosis contributes to resistance against chemotherapeutic agents [49, 50]. Investigating the interplay between deubiquitinating enzymes (DUBs) and ferroptosis in CRC may offer valuable insights into cancer progression and therapeutic vulnerabilities [51, 52]. The role of USP5 in regulating protein turnover and stability is crucial for cellular homeostasis, influencing multiple cellular processes [33].

Our findings confirm that USP5 does not carry out its traditional function as a deubiquitinating enzyme in the regulation of CRC initiation and progression. Instead, it facilitates the lysosomal degradation of YBX3 (Y-box binding protein 3), and through the USP5/YBX3/SLC7A11 axis, it modulates CRC progression by coordinating autophagy and ferroptosis.

## Result

### USP5 knockout sensitizes CRC cells to ferroptosis

**Fig. 1:**
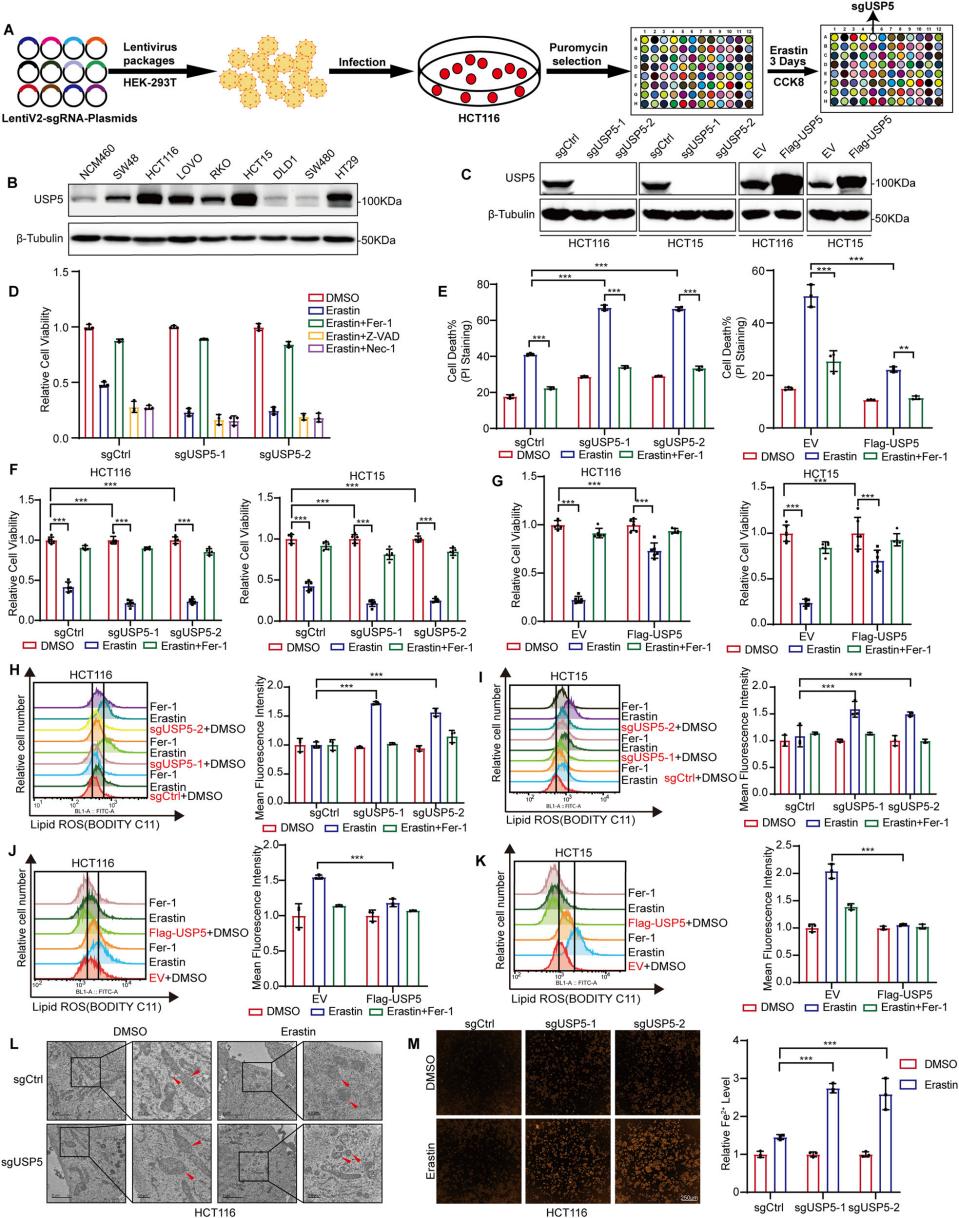
USP5 knockout sensitizes CRC cells to ferroptosis. **A** Constructed a deubiquitinating enzyme knockout gene library in colorectal cancer cells, treated with Erastin (15 µM) for 4 days (72 h), and measured cell viability using the CCK-8 assay. **B** Compared USP5 protein levels in the normal human colon epithelial cell line (NCM460) and eight CRC cell lines. **C** Assessed USP5 knockout and overexpression efficiency in HCT116 and HCT15 cells using western blot. **D** Following Erastin treatment, USP5 knockout cells were subjected to rescue treatments with Fer-1 (5 µM), Z-VAD (10 µM), and Nec-1(10 µM). Cell viability was subsequently measured using the Cell Counting Kit-8 (CCK-8) assay (*n* = 3). **E** USP5 knockout and USP5-overexpressing cells were treated with Erastin(15/20 µM) and Fer-1(5 µM), followed by PI staining after 48 hours. Cell viability was assessed by flow cytometry (*n* = 3). **F**, **G** In HCT116 and HCT15 cell lines, USP5 knockout cells were treated with Erastin (15 µM) for 72 h, while USP5 overexpressing cells received Erastin (20 µM) under the same conditions. Cell viability was subsequently assessed using the CCK-8 assay (*n* = 6). **H**, **I** Lipid-ROS levels in USP5 knockout cell lines were analyzed via flow cytometry following Erastin (15 µM) treatment and Fer-1 (5 µM) rescue, with corresponding quantitative analysis of Lipid-ROS levels. **J**, **K** Flow cytometry was employed to assess Lipid-ROS levels in USP5 overexpressing cell lines after Erastin (20 µM) treatment and Fer-1(5 µM) rescue, followed by quantitative analysis of Lipid-ROS levels (*n* = 3). **L** Representative TEM micrographs of sgCtrl and sgUSP5 HCT116 cells before and after treatment with Erastin. **M** Intracellular Fe^2+^ levels were determined using ferroOrnge in USP5 knockout cells with quantitative analysis presented (*n* = 3). Data are shown as means ± SD. *P < 0.05, **P < 0.01, ***P < 0.001, one- way ANOVA and two-way ANOVA.

To investigate the role of deubiquitination in the regulation of ferroptosis in CRC we systematically screened 108 deubiquitinases (DUBs) to identify key regulatory factors and elucidate their underlying mechanisms. Two pairs of sgRNAs were designed for each DUB, and corresponding CRISPR-Cas9 vectors were constructed to establish a comprehensive knockout library. These vectors were transfected into CRC cell lines, resulting in the generation of 108 distinct knockout clones, collectively forming a comprehensive DUB knockout panel. Both control and DUB-deficient cell lines were treated with the ferroptosis inducer Erastin, and cell viability was measured to identify candidates whose deletion impaired survival (Fig. 1A). This screen identified USP5 as a potential negative regulator of ferroptosis. Western blot analysis revealed that USP5 expression was markedly upregulated in most CRC cell lines compared to the normal colorectal epithelial cell line NCM460 (Fig. 1B). To further investigate its role, we established USP5-overexpressing and USP5-knockout models in HCT116 and HCT15 cells (Fig. 1C).

We first determined the IC_50_ of Erastin in colorectal cancer cells (15.01 μM) to ensure biological relevance of the concentration used in subsequent experiments (Fig. S1A). To clarify the specific regulatory role of USP5 knockout in ferroptosis, this study co-treated Erastin-exposed USP5-knockout cells with ferroptosis inhibitor Ferrostatin-1 (Fer-1), apoptosis inhibitor Z-VAD-FMK (Z-VAD), and necroptosis inhibitor Necrostatin-1 (Nec-1). The results demonstrated that only the Fer-1 treatment group exhibited significant recovery of cell viability, confirming the specific association between USP5 deficiency and the ferroptosis pathway through this selective rescue effect (Fig. 1D). Propidium iodide (PI) staining was utilized to assess cell death in USP5-knockout cells treated with Erastin. Flow cytometry analysis further confirmed that cells became more sensitive to Erastin following USP5 knockout (Fig. 1E). Functional assays validated by microscopic viability assessment and CCK-8 (Cell Counting Kit-8) assays showed that USP5 knockout sensitized colorectal cancer cells to ferroptosis, significantly reducing cell viability after 72 hours of Erastin treatment, with cell viability significantly restored by Fer-1 (5 μM). In contrast, USP5 overexpression conferred resistance to ferroptosis (Fig. 1F, G and Fig. S1B, C). Mechanistically, flow cytometric analysis showed that USP5 deficiency led to increased lipid peroxidation and accumulation of intracellular lipid reactive oxygen species (ROS) following Erastin treatment (Fig. 1H, I). Conversely, USP5 overexpression reduced these effects by lowering lipid ROS levels (Fig. 1J, K). Transmission electron microscopy revealed classical ferroptotic ultrastructural changes in USP5-knockout cells, including rupture of the outer mitochondrial membrane, reduced mitochondrial volume, and loss or disorganization of cristae (Fig. 1L). FerroOrange staining confirmed that Erastin-treated USP5-knockout cells exhibited marked accumulation of ferrous iron (Fe²⁺), which further exacerbated ferroptosis (Fig. 1M). Conversely, USP5-overexpressing cells showed reduced Fe²⁺ levels compared to wild-type counterparts (Fig. S1D).

Collectively, these findings identify USP5 as a critical negative regulator of ferroptosis in CRC. Its loss promotes ferroptotic sensitivity through enhanced lipid peroxidation, mitochondrial damage, and iron overload.

### High USP5 expression promotes proliferation and migration of CRC

**Fig. 2:**
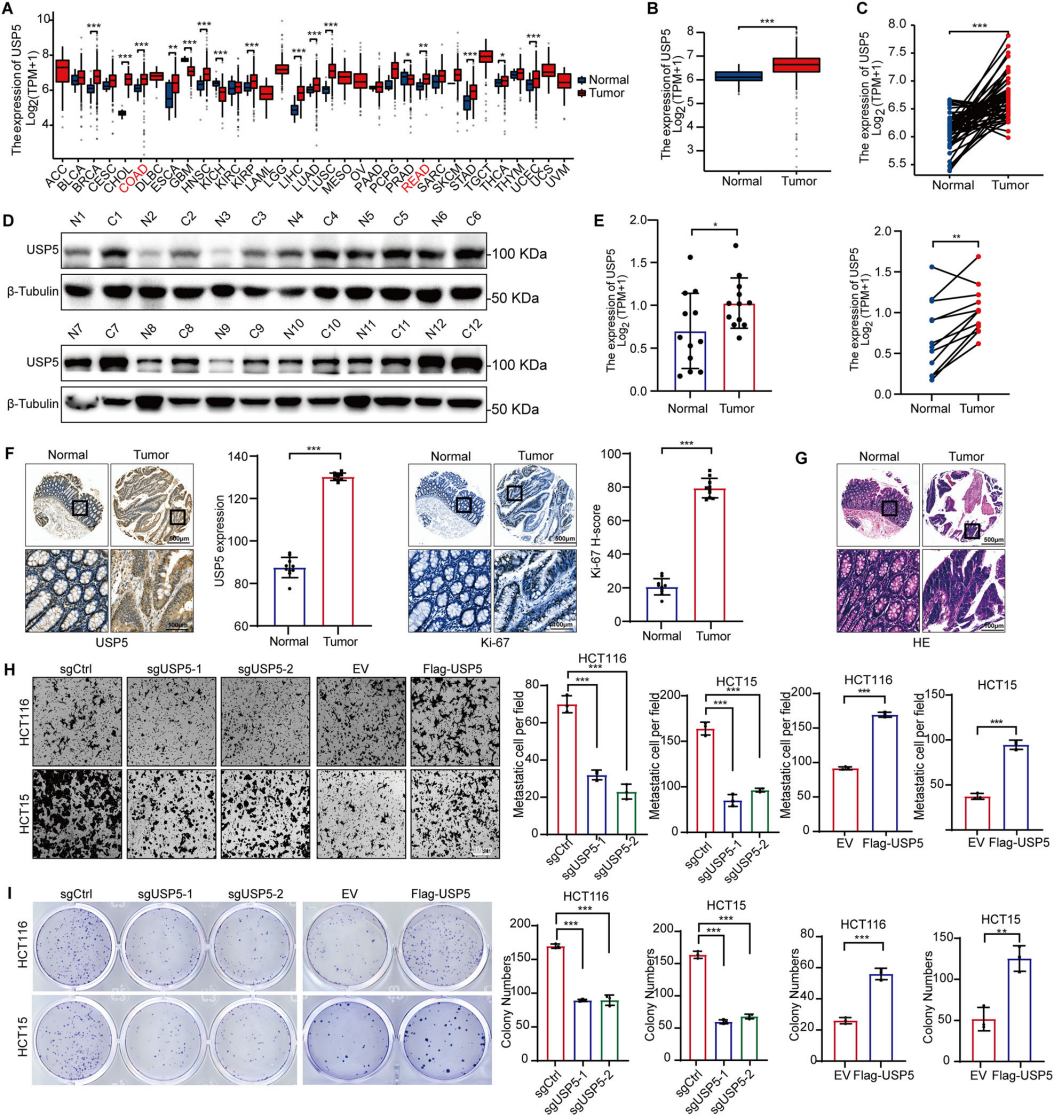
High USP5 expression promotes proliferation and migration of CRC. **A–C** Analyzed and compared USP5 expression levels across different cancers using databases such as TCGA, including comparisons between tumor and adjacent normal tissues. **D**, **E** Measured and visualized USP5 expression levels in tumor and adjacent normal tissues from 12 patient pairs. **F** Representative images of immunohistochemical (IHC) staining for USP5 and Ki-67 in normal and tumor colorectal tissues (left), with corresponding quantification (right) (*n* = 10). **G** Representative hematoxylin and eosin (H&E) staining images of normal and tumor colorectal tissues. **H** Conducted transwell chamber assays for USP5 knockout and overexpression groups with corresponding data analysis (*n* = 3). **I** Performed colony formation assays for USP5 knockout and overexpression groups, with statistical analysis of the results (*n* = 3). Data are shown as means ± SD. *P < 0.05, **P < 0.01, ***P < 0.001, unpaired Student´s *t* test, one-way ANOVA, and two-way ANOVA.

To investigate the role of USP5 in CRC, we performed comprehensive bioinformatics analyses to assess its expression profile. Data from The Cancer Genome Atlas (TCGA) revealed that USP5 is broadly overexpressed across various cancer types, including CRC, with elevated levels observed in both tumor tissues and adjacent non-tumor tissues (Fig. 2A–C). Analysis of 12 paired CRC tumor and adjacent normal tissues further confirmed significantly higher USP5 expression in most tumor samples (Fig. 2D, E). Immunohistochemistry (IHC) analysis demonstrated markedly increased USP5 expression in tumor tissues compared to adjacent normal tissues. Notably, Ki-67, a marker of cellular proliferation, was also elevated in tumor sections (Fig. 2F). Histopathological assessment using hematoxylin and eosin (H&E) staining revealed malignant features in tumor tissues, including disorganized architecture and increased cellularity (Fig. 2G). Functional assays provided further evidence for the oncogenic role of USP5. Transwell migration assays showed that USP5 overexpression significantly promoted cell migration (Fig. 2H). USP5 elevation enhanced CRC cell proliferation as measured by CCK-8 assays, whereas USP5 knockout attenuated such effects (Fig. S2A, B). Furthermore, colony formation assays revealed that USP5 overexpression significantly increased proliferative capacity in CRC cells, confirming its pro-tumorigenic function in colorectal carcinogenesis (Fig. 2I). Notably, treatment of USP5-knockout cell lines with the ferroptosis inhibitor Fer-1 partially restored their growth, suggesting that USP5 may regulate CRC cell proliferation, at least in part, by modulating ferroptosis-related pathways (Fig. S2C).

These findings highlight the critical role of USP5 in promoting CRC cell proliferation, migration, and overall tumor progression.

### USP5 regulates ferroptosis by stabilizing SLC7A11

**Fig. 3:**
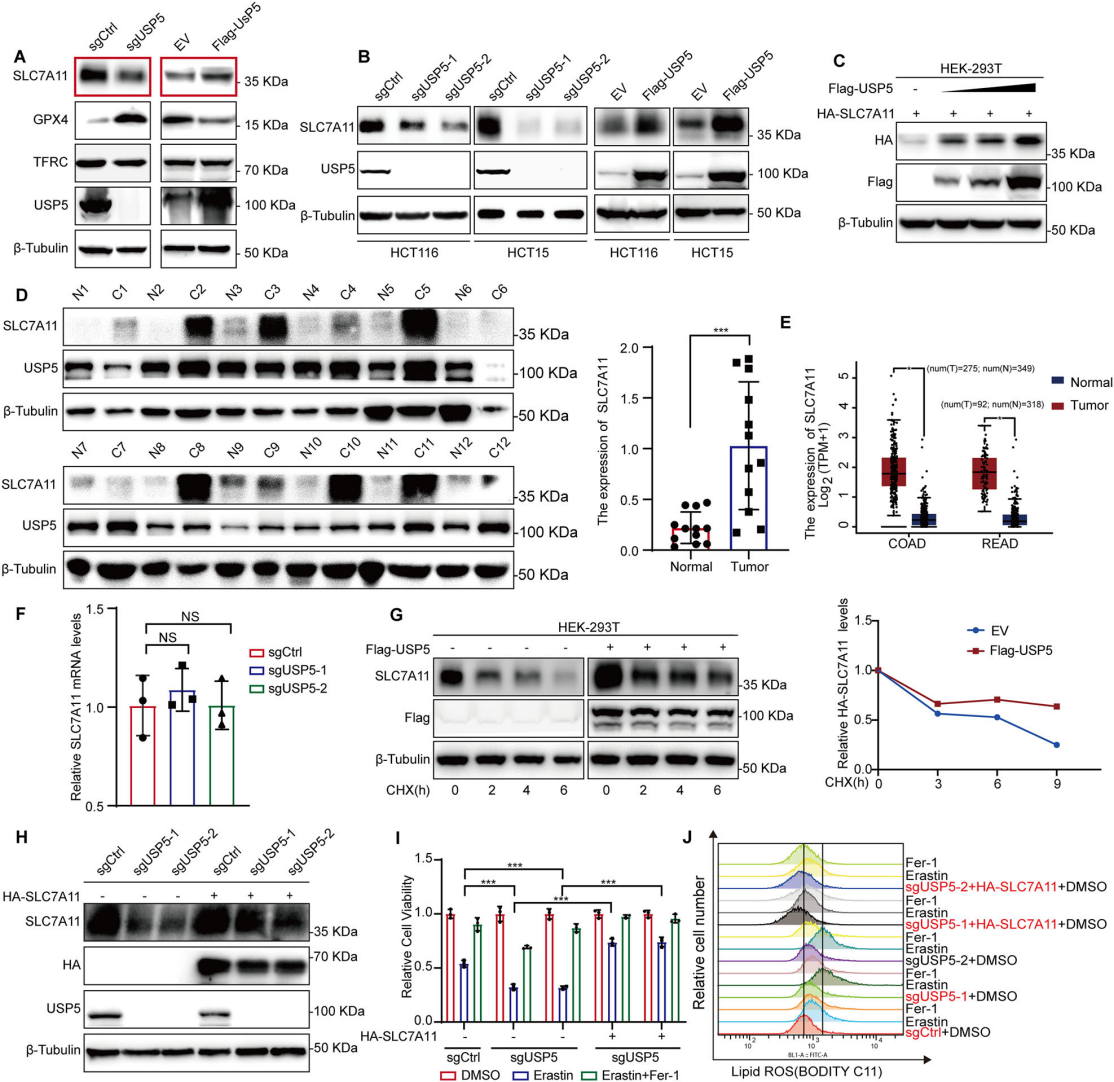
USP5 regulates ferroptosis by stabilizing SLC7A11. **A** Western blot analysis of ferroptosis-related protein expression following USP5 knockout or overexpression. **B** Confirmation of SLC7A11 expression changes in sgCtrl/sgUSP5 and EV/ Flag-USP5 HCT116 or HCT15 cell lines. **C** Transient transfection of 0–1 μg USP5 plasmid and 0.25 μg HA-SLC7A11 plasmid into HEK-293T cells, followed by protein expression analysis 48 h later. **D** Western blot analysis of SLC7A11 expression in cancer and adjacent normal tissues from 12 patient samples, focusing on cases with high USP5 expression, with corresponding data analysis (*n* = 12). **E** Box plots showing SLC7A11 mRNA expression levels in colorectal adenocarcinoma (COAD) and rectal adenocarcinoma (READ) based on TCGA datasets, comparing tumor and normal samples. **F** Using qPCR technology to detect the mRNA levels of SLC7A11 in the control group and USP5 knockout group (*n* = 3). **G** Transfect HEK-293T cells with Flag-USP5. After 24 h of transfection, treat the cells with CHX (100 μg/mL) for the specified time points. Analyze SLC7A11 protein levels using Western blot. **H** In USP5 knockout cell lines, transiently transfect HA-SLC7A11 and assess its expression using western blot analysis. **I** Cell viability assay in the sgCtrl and USP5 knockout cells after SLC7A11 reconstitution under different treatment conditions (DMSO, Erastin (15 µM), Erastin and Fer-1 (5 µM)) (*n* = 3). **J** Flow cytometric analysis of lipid ROS levels using BODIPY-C11 staining under the indicated conditions. Data are shown as means ± SD. *P < 0.05, **P < 0.01, ***P < 0.001, unpaired Student´s *t* test, one-way ANOVA, and two-way ANOVA.

To delineate the mechanism by which USP5 regulates ferroptosis, we examined key components of the ferroptotic pathway in both USP5-knockout and USP5-overexpressing cells. The expression of SLC7A11, a core subunit of the cystine/glutamate antiporter system Xc⁻, was significantly modulated following USP5 alteration, a trend consistently observed across multiple cell lines (Fig. 3A, B). In 293 T cells, dose-dependent transfection with Flag-tagged USP5 resulted in a progressive increase in SLC7A11 protein levels, indicating that USP5 promotes SLC7A11 accumulation (Fig. 3C). In patient-derived CRC samples, tumors with high USP5 expression displayed significantly elevated SLC7A11 levels (Fig. 3D). TCGA analysis further confirmed that SLC7A11 is upregulated in both colon and rectal cancers (Fig. 3E). Additionally, analysis of the TCGA database suggests a potential association between USP5 and SLC7A11 (Fig. S3A). Next, we examined the SLC7A11 mRNA expression levels in the sgCtrl group and the USP5 knockout groups (sgUSP5-1 and sgUSP5-2). The results showed that there were no significant differences in SLC7A11 mRNA levels between the sgCtrl group and the USP5 knockout groups (Fig. 3F). Cycloheximide (CHX) chase assays demonstrated that USP5 overexpression delayed CHX-induced degradation of SLC7A11, suggesting that USP5 stabilizes the SLC7A11 protein (Fig. 3G). To assess functional relevance, we reintroduced SLC7A11 into USP5-deficient cells. Western blot analysis confirmed partial restoration of SLC7A11 expression (Fig. 3H). Upon treatment with Erastin and the ferroptosis inhibitor Fer-1, SLC7A11 overexpression significantly attenuated USP5-mediated ferroptotic cell death and lipid ROS accumulation, as determined by flow cytometry and CCK-8 assays (Fig. 3I, J).

These findings identify USP5 as a negative regulator of ferroptosis through stabilization of SLC7A11, thereby supporting CRC cell survival and tumor progression.

### USP5 promotes lysosomal degradation of YBX3

**Fig. 4:**
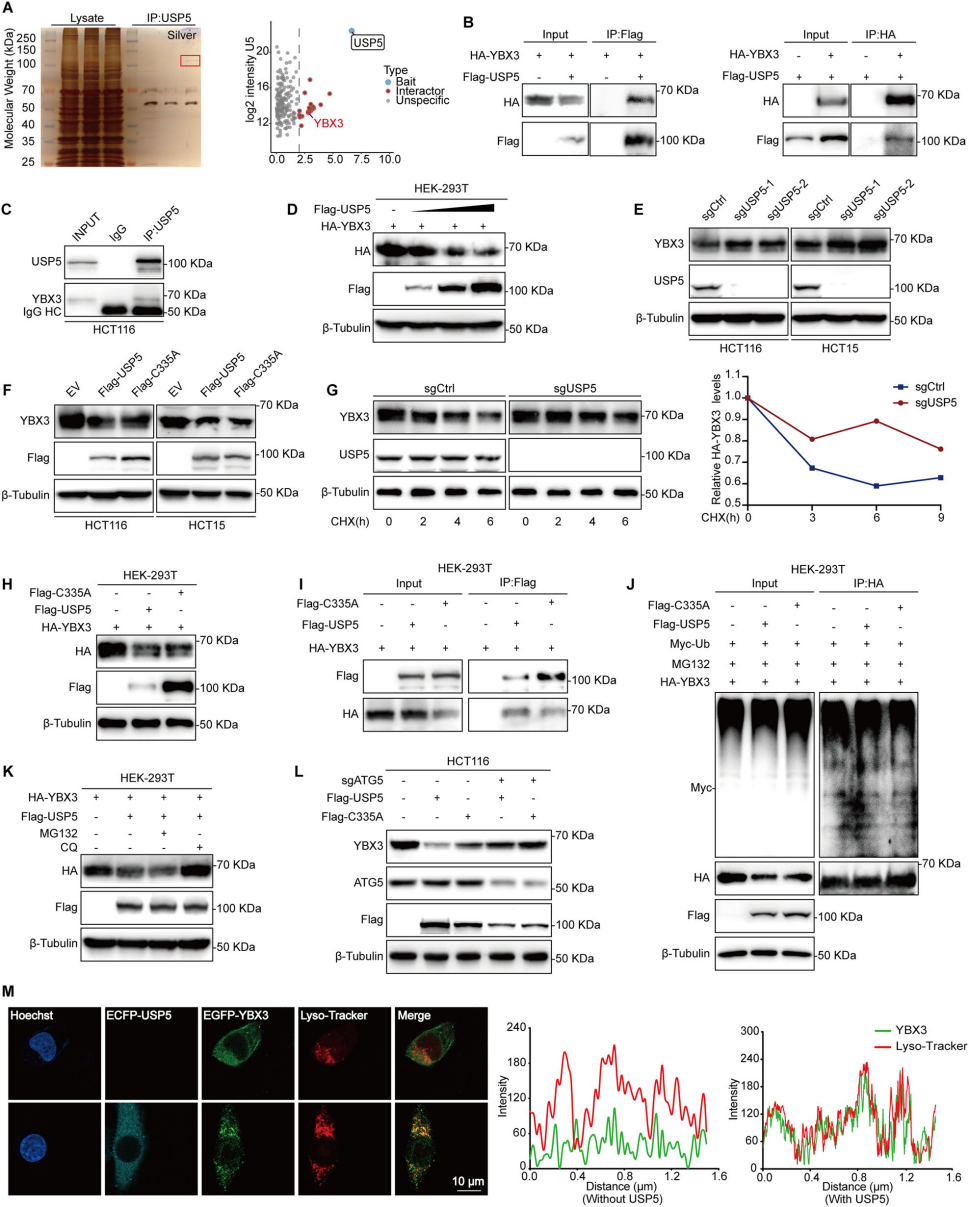
USP5 promotes lysosomal degradation of YBX3. **A** Silver staining and mass spectrometry analysis of proteins co-immunoprecipitated with USP5. HCT116 cell lysates were immunoprecipitated using anti-USP5 antibody. Highlighted regions indicate USP5 and YBX3. Right panel: Volcano plot showing proteins enriched in USP5 immunoprecipitates. **B** Co-immunoprecipitation of HA-YBX3 and Flag-USP5 in HEK-293T cells. Left: Flag immunoprecipitation; Right: HA immunoprecipitation. **C** Endogenous interaction between USP5 and YBX3 validated by co-immunoprecipitation in HCT116 cells. IgG HC: IgG Heavy Chain. **D** Transiently transfect 0-2 μg Flag-USP5 plasmid and 0.5 μg HA-YBX3 plasmid into HEK-293T cells, collect cells 48 hours later for protein expression analysis. **E** Western blot to analyze YBX3 expression in HCT116 and HCT5 cells following USP5 knockout. **F** Western blot to analyze YBX3 expression in HCT116 and HCT5 cells following USP5 overexpression. **G** Western blotting analysis exhibiting YBX3 remaining level at indicated time in HCT16 with USP5 knockout and treatment with CHX (100 μg/mL), a protein synthesis inhibitor. **H** Co-transfect HA-YBX3 with Flag-USP5 and Flag-C335A (enzyme activity mutant) into HEK293T cells for 48 h, then detect HA-YBX3 expression. **I** Transfect Flag-USP5 and Flag-C335A into 293 T cells, collect cells 48 h later for Co-IP. **J** Transfect three plasmid groups into 293 T cells, treat with MG132 for 12 h after 36 hours of transfection, and perform denaturing Co-IP. **K** Transfect Flag-USP5 and HA-YBX3 into 293 T cells, add MG132 or CQ to co-transfected groups, and perform western blot to analyze protein expression. **L** Transfect Flag-USP5, Flag-C335A, and Lenti-sgATG5 into 293 T cells, and use western blot to analyze YBX3 protein levels. **M** Set up two experimental groups: one group will be transfected with ECFP-USP5 and EGFP-YBX3, while the other group will not be transfected with ECFP-USP5. After 48 h of culture, stain the lysosomes using the Lyso Tracker^TM^ probe and analyze the samples using confocal microscopy. Nucleus were stained with Hoechst. Scale bar, 10 μm.

To uncover the mechanism by which USP5 regulates ferroptosis, we initially identified SLC7A11 as a potential downstream target through preliminary screening experiments. However, co-immunoprecipitation (Co-IP) analysis revealed that USP5 does not directly interact with SLC7A11 (Fig. S4A). To further explore the regulatory network, we performed silver staining followed by mass spectrometry, which identified YBX3 as a novel interacting protein of USP5 (Fig. 4A). The interaction between USP5 and YBX3 was confirmed by both exogenous co-expression of Flag- and HA-tagged constructs and endogenous Co-IP assays in HCT116 cells, demonstrating a strong and specific protein–protein association (Fig. 4B, C). Domain-mapping experiments indicated that all functional domains of USP5 are capable of binding to YBX3 (Fig. S4B).

To investigate the regulatory impact of USP5 on YBX3 expression, dose-dependent co-transfection experiments in 293 T cells demonstrated that increasing USP5 expression resulted in a progressive reduction in YBX3 protein levels, suggesting that USP5 promotes YBX3 degradation (Fig. 4D). Consistently, endogenous USP5 knockout led to a marked upregulation of YBX3, whereas overexpression suppressed its levels, further supporting a negative regulatory role of USP5 on YBX3 (Fig. 4E, F). CHX chase assays revealed that USP5 shortens the half-life of YBX3 and accelerates its degradation (Fig. 4G and Fig. S4C). Notably, both wild-type USP5 and its catalytically inactive mutant (C335A, in which the catalytic cysteine at position 335 is substituted with alanine) promoted YBX3 degradation, indicating that this regulatory effect is independent of USP5’s canonical deubiquitinase activity (Fig. 4H). Co-IP assays confirmed that both wild-type and mutant USP5 retain their ability to interact with YBX3, further demonstrating that enzymatic activity is not required for this interaction (Fig. 4I). Additionally, ubiquitin immunoprecipitation (IP) assays showed no significant differences in YBX3 ubiquitination levels between cells expressing wild-type and mutant USP5, suggesting that USP5 does not modulate YBX3 degradation through a ubiquitin-dependent mechanism (Fig. 4J).

To define the degradation pathway, cells were treated with the proteasome inhibitor MG132 or the lysosome inhibitor Chloroquine (CQ). Only CQ treatment restored YBX3 protein levels, indicating that USP5 mediates YBX3 degradation via the lysosomal pathway (Fig. 4K). Furthermore, knockdown of ATG5, a core autophagy-related gene, rescued YBX3 levels in USP5-overexpressing cells (Fig. 4L). These results suggest that USP5 promotes the degradation of YBX3 through regulation of the autophagy-lysosomal pathway. To further validate the involvement of the lysosomal pathway, we performed immunofluorescence analysis. The results showed that USP5 facilitates the co-localization of YBX3 with lysosomes, providing additional evidence to support the conclusion that YBX3 is degraded through a USP5-dependent lysosomal pathway (Fig. 4M and Fig. S4D)

These findings identify YBX3 as a novel downstream effector of USP5 and reveal a non-canonical, lysosome-mediated mechanism by which USP5 regulates ferroptosis.

### YBX3 regulates cellular ferroptosis by lysosomal degradation of SLC7A11

**Fig. 5:**
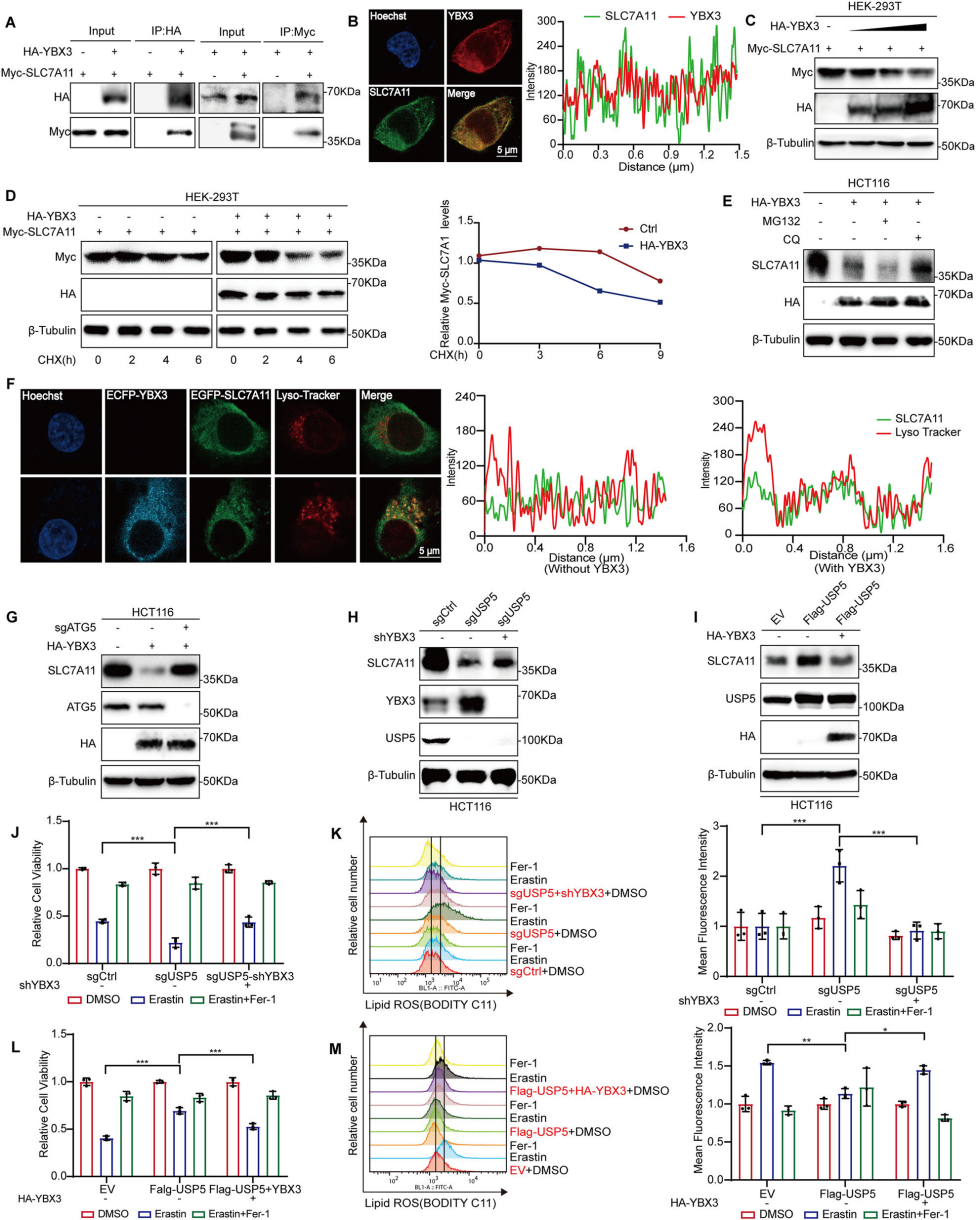
YBX3 regulates cellular ferroptosis by lysosomal degradation of SLC7A11. **A** Co-immunoprecipitation assays showing interaction between HA-YBX3 and Myc-SLC7A11 in HEK-293T cells. Cell lysates were immunoprecipitated with anti-HA or anti-Myc antibodies and analyzed by western blotting using indicated antibodies. **B** Confocal images showing the co-localization of HA-YBX3 and Myc-SLC7A11 in HEK-293T cells. HA-YBX3 is labeled in red, Myc-SLC7A11 is labeled in green, and the merged image shows their overlap. Scale bar: 5 µm. **C** Transiently transfect 0–2 μg HA-YBX3 plasmid and 0.5 μg Myc-SLC7A11 plasmid into HEK-293T cells, collect cells 48 hours later for protein expression analysis. **D** CHX chase assay assessing Myc-SLC7A11 protein stability in the presence or absence of HA-YBX3 overexpression in HEK-293T cells. Protein levels were measured at indicated time points and quantified (right panel). **E** HEK-293T cells transfected with HA-YBX3 were treated with proteasome inhibitor MG132 or lysosomal inhibitor CQ, followed by western blot analysis of SLC7A11 expression. **F** Set up two experimental groups: one group will be transfected with ECFP-YBX3 and EGFP-SLC7A11, while the other group will not be transfected with ECFP-YBX3. After 48 hours of culture, stain the lysosomes using the Lyso Tracker^TM^ probe and analyze the samples using confocal microscopy. Nucleus were stained with Hoechst. Scale bar, 5 μm. **G** Detect the protein expression level of SLC7A11 after transient overexpression of YBX3 and transient knockdown of ATG5 in cells. **H** Western blot analysis of SLC7A11 and YBX3 levels in HCT116 cells with stable USP5 knockout and shYBX3. **I** In USP5 overexpressing cells, transiently overexpress YBX3 and detect the protein level of SLC7A11. **J** Cell viability measured by CCK-8 assay in sgCtrl, sgUSP5 and sgUSP5+shYBX3 HCT116 cells, treated with DMSO, Erastin (15 μM), or Erastin + Fer-1 (5 μM) for 48 h (*n* = 3). **K** Using BODIPY-C11 staining and flow cytometry to assess lipid peroxidation (lipid ROS) levels in three treatment groups; right panel shows quantification of mean fluorescence intensity (*n* = 3). **L** Cell viability measured by CCK-8 assay in EV, Flag-USP5 and F lag-USP5 + HA-YBX3 HCT116 cells, treated with DMSO, Erastin (20 μM), or Erastin+Fer-1 (5 μM) for 48 h (*n* = 3). **M** Using BODIPY-C11 staining and flow cytometry to assess lipid peroxidation (lipid ROS) levels in three treatment groups; right panel shows quantification of mean fluorescence intensity (*n* = 3). Data are shown as means ± SD. *P < 0.05, **P < 0.01, ***P < 0.001, two-way ANOVA.

Building on previous findings, this study further explored the relationship between YBX3 and SLC7A11 to clarify the role of YBX3 in the regulation of ferroptosis. Co-IP assays using ectopically expressed HA-YBX3 and Myc-SLC7A11 revealed a physical interaction between the two proteins (Fig. 5A). Building on this, we employed immunofluorescence confocal microscopy to examine the spatial colocalization of YBX3 and SLC7A11. The results confirmed that the two proteins exhibit significant colocalization within the cell, indicating a close spatial association between them (Fig. 5B and Fig. S5A). To explore the regulatory impact of YBX3 on SLC7A11 expression, we performed dose-dependent co-transfection assays. Increasing amounts of HA-YBX3 plasmid led to a progressive reduction in Myc-SLC7A11 protein levels, indicating that YBX3 promotes SLC7A11 degradation (Fig. 5C). CHX chase assays further confirmed that overexpression of YBX3 significantly accelerated SLC7A11 degradation (Fig. 5D). To dissect the degradation pathway involved, HCT116 cells overexpressing HA-YBX3 were treated with either MG132 or CQ. Notably, CQ treatment–but not MG132–restored SLC7A11 protein levels, suggesting that YBX3 facilitates lysosome-dependent degradation of SLC7A11 (Fig. 5E).

Building on this discovery, immunofluorescence confocal microscopy demonstrated YBX3-mediated regulation of SLC7A11 lysosomal accumulation and subsequent degradation (Fig. 5F and Fig. S5B). Transient overexpression of YBX3 followed by ATG5 knockout resulted in partial restoration of SLC7A11 expression compared to YBX3-overexpressing controls, thereby providing mechanistic evidence that YBX3 regulates SLC7A11 through the autophagic pathway (Fig. 5G).

The biological relevance of this regulatory axis was examined in USP5 knockout cells. Knockdown of YBX3 (shYBX3) in this context markedly restored SLC7A11 protein levels compared to USP5 knockout alone (Fig. 5H). In HCT116 cells overexpressing USP5, transient expression of HA-YBX3 reversed the USP5-mediated stabilization of SLC7A11, further confirming the antagonistic role of YBX3 (Fig. 5I). Functional assays revealed that YBX3 knockdown significantly rescued the ferroptosis sensitivity of USP5-deficient cells, as demonstrated by improved cell viability upon Erastin treatment (Fig. 5J). Consistently, BODIPY-C11 staining coupled with flow cytometry showed reduced lipid ROS accumulation in the double-knockdown group, highlighting YBX3’s role in promoting ferroptosis (Fig. 5K). Conversely, in USP5-overexpressing cells, reintroduction of YBX3 impaired the protective effect of USP5, as indicated by reduced cell viability and enhanced lipid peroxidation upon Erastin exposure (Fig. 5L, M).

These findings demonstrate that YBX3 negatively regulates SLC7A11 through lysosome-mediated degradation, and highlight the critical role of the USP5–YBX3–SLC7A11 axis in orchestrating ferroptosis by modulating SLC7A11 stability, cell viability, and lipid ROS accumulation.

### Organoid and xenograft models confirm USP5’s role in regulating ferroptosis and CRC progression

To explore the role of USP5 in patient-derived CRC tissues and its influence on ferroptosis in a clinically relevant context, we established CRC organoids from primary tumor samples of a CRC patient. These organoids, which retain the genetic, molecular, and phenotypic features of the original tumor, provide a robust ex vivo platform to assess the functional impact of USP5 depletion on ferroptosis susceptibility and tumor growth dynamics.

**Fig. 6:**
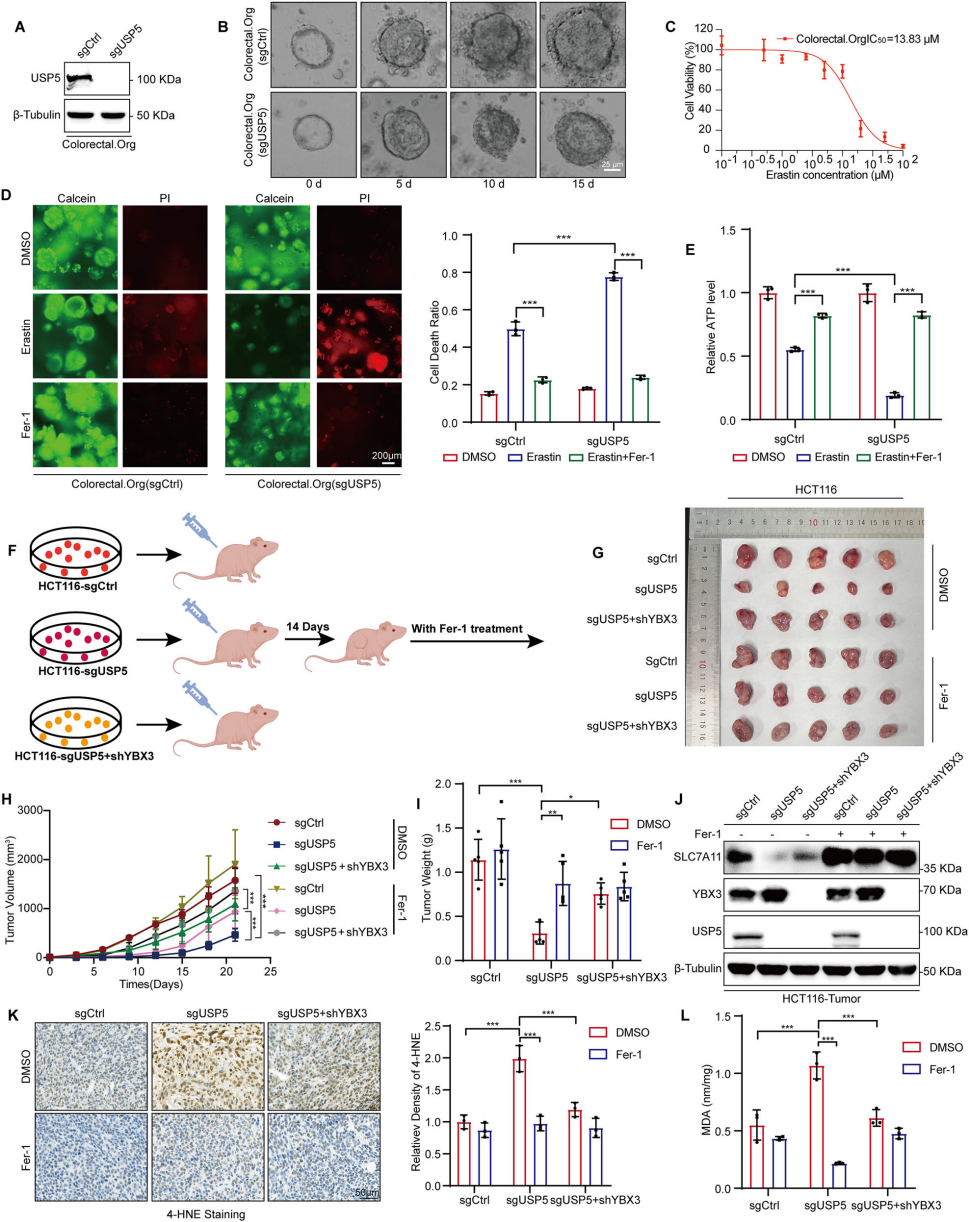
Organoid and xenograft models confirm USP5’s role in regulating ferroptosis and CRC progression. **A** Knockout efficiency of USP5 in colorectal organoids was confirmed by western blot assays. **B** sgCtrl and sgUSP5 colorectal organoids were visualized by microscope at 5-day intervals. **C** Determination of IC_50_ in normal human colorectal cancer organoids. Scale bar, 25 μm. **D** sgCtrl and sgUSP5 colorectal organoids were treated with DMSO, Erastin (15 μM) and Erastin + Fer-1 (5 μM) for 48 h. Organoids were stained using the Calcein/PI Cell Viability Kit and visualized by fluorescence microscope. **E** Organoid viability was quantitatively assessed using the ATP Assay Kit. **F**–**I** Therapy of Fer-1 promotes xenograft tumor growth in USP5 knockout cells and recovery tumor growth and weight by Fer-1(0.5 mg/mL), tumor volume, and tumor weight in mice (*n* = 5). **J** Expression of SLC7A11 in xenograft tumor by western blot assays. **K** Detection of 4-HNE Levels in Different Tumor Types Treated with DMSO or Fer-1 (*n* = 3). **L** Detection of MDA content in different tumors treated with DMSO and the Fer-1 combination (*n* = 3). Data are shown as means ± SD. ****P < 0.0001, unpaired Student´s *t* test or two-way ANOVA.

USP5-knockout (sgUSP5) organoids were generated via CRISPR/Cas9 gene-editing, and USP5 depletion was confirmed by western blot (Fig. 6A). Growth assays revealed that the loss of USP5 significantly impaired organoid proliferation compared to control organoids (sgCtrl) (Fig. 6B). The sensitivity to Erastin was assessed by determining the half-maximal inhibitory concentration (IC₅₀) (Fig. 6C). Subsequently, organoids were treated with 15 μM Erastin for 72 hours. Compared to controls, sgUSP5 organoids showed significantly decreased Calcein-AM staining (viable cell marker), increased PI staining (cell death marker), and markedly reduced ATP levels, indicating enhanced ferroptotic cell death upon USP5 knockout. These effects could be reversed by Fer-1 (Fig. 6D, E). To evaluate the physiological relevance of USP5-mediated ferroptosis regulation in vivo, we employed a xenograft tumor model. Nude mice were subcutaneously injected with sgCtrl HCT116, sgUSP5 HCT116, and sgUSP5 HCT116 cells with stable knockdown of YBX3 (sgUSP5 + shYBX3). In the in vivo model, we did not treat the subcutaneously injected cells with Erastin but instead used Fer-1, in order to specifically validate the intrinsic biological function of USP5 knockout and its unique role in the USP5–YBX3–SLC7A11 regulatory axis. Each group was further subdivided for treatment with control (DMSO) or Fer-1 (Fig. 6F). Tumor volumes were monitored throughout the experiment, and excised tumors were weighed at the endpoint (Fig. 6G–I). Notably, Fer-1 treatment significantly restored tumor volume and mass in the sgUSP5 group, suggesting that ferroptosis inhibition rescued the growth-suppressive effects induced by USP5 deletion. Correspondingly, western blot analysis revealed reactivation of SLC7A11 expression in tumors from the Fer-1-treated sgUSP5 group (Fig. 6J).

To further verify the involvement of ferroptosis in USP5-mediated tumor regulation, we measured the expression levels of two key markers of lipid peroxidation and ferroptosis—4-hydroxynonenal (4-HNE) and malondialdehyde (MDA)—in tumor tissues from each experimental group. The results showed that USP5 knockout significantly increased the expression level of 4-HNE, while treatment with Fer-1 effectively suppressed this elevation, further confirming the participation of ferroptosis in this process (Fig. 6K). Subsequent analysis of MDA also indicated that USP5 knockout led to a significant increase in MDA levels in tumor tissues, whereas Fer-1 treatment markedly reduced this increase (Fig. 6L).

Collectively, these findings demonstrate that USP5 depletion sensitizes CRC cells to ferroptosis and suppresses tumor progression in both patient-derived organoid and in vivo xenograft models, highlighting the USP5/YBX3-ferroptosis axis as a potential therapeutic target in CRC.

## Discussion

Ferroptosis has emerged as a breakthrough in cancer therapy, profoundly affecting cancer cell physiology. Previous studies have shown that USP5 can target LSH to resist ferroptosis in liver cancer cells and promote the malignant transformation of tumors [50]. This study systematically investigates the role of USP5 in the progression of CRC and suggests that USP5 regulates ferroptosis resistance in CRC by lysosome-dependent degradation of YBX3, which in turn stabilizes the expression of SLC7A11. Although USP5 is widely recognized as a typical oncogene, its function and significance in CRC are yet to be clarified.

In this study, we systematically screened 108 deubiquitinases (DUBs) and identified USP5 as a key regulatory factor of ferroptosis in CRC cells. Our research positions USP5 as a molecular regulator that balances cell survival and ferroptosis in CRC cells. Previous studies have indicated that USP5 plays an important role in cancer progression. For example, in breast cancer, USP5 promotes tumor progression by stabilizing HIF-2α, while in non-small cell lung cancer, USP5 enhances immune escape through PD-L1, promoting cancer development [34, 35]. Furthermore, unlike other DUBs [23, 53], the deletion of USP5 significantly increased lipid peroxidation, mitochondrial membrane rupture, and Fe²⁺ accumulation when treated with Erastin, suggesting that USP5 regulates ferroptosis through a specific mechanism in CRC cells.

The high expression of USP5 in CRC tissues and its role in promoting cell proliferation and migration indicate its multifunctional oncogenic properties. Notably, the ferroptosis inhibitor Fer-1 partially restores cell growth in USP5 knockout cells, suggesting that USP5 not only acts through classical oncogenic pathways but also regulates the balance between tumor cell survival and death by modulating ferroptosis [34, 36].

Traditionally, DUBs stabilize substrate proteins through deubiquitination [54]. For example, MSK1 increases Snail protein stability through USP5-mediated deubiquitination of Snail, promoting CRC metastasis [55]. Studies have also shown that USP5 can promote the K48-linked polyubiquitination of NLRP3 via recruitment of the E3 ligase MARCHF7 and mediate its degradation in autophagy, inhibiting the inflammasome signaling pathway [56]. This study further confirms the regulatory role of USP5 in autophagy. It is worth noting that the functional studies of USP5 are rapidly expanding, with its regulatory network involving DNA repair, inflammation, tumorigenesis, neurodegenerative diseases, and other areas [35]. By dynamically balancing the stability and degradation of substrate proteins, USP5 reveals its multifunctional regulatory hub role in complex pathological networks such as cancer and inflammation, providing new perspectives for disease-targeted therapy.

YBX3 is involved in cellular stress responses and gene regulation, playing a crucial role in modulating key signaling pathways and stress-related mechanisms [43, 57]. In hepatocellular carcinoma (HCC), YBX3 promotes tumor growth by enhancing cell survival under stress conditions through the regulation of oxidative stress responses and metabolic reprogramming [58, 59]. Closely associated with cell death mechanisms like autophagy, YBX3 also influences chemoresistance in breast cancer by affecting autophagy-related pathways [60, 61].

SLC7A11, as the core subunit of the system Xc⁻, plays a pivotal role in determining a cell’s sensitivity to ferroptosis [17, 18]. It is noteworthy that ferroptosis has a dual role in various cancer types: it can inhibit tumor growth through its cytotoxic effects or promote tumor survival through adaptive responses [16, 19, 62–64]. Dysregulation of iron metabolism is common in CRC, making cancer cells more sensitive to ferroptosis [65–67]. This study found that USP5 stabilizes SLC7A11 and enhances its protein levels. Importantly, USP5’s regulation of SLC7A11 does not depend on direct interaction, but rather occurs indirectly through the downstream effector molecule YBX3. In contrast, in liver cancer, USP5 stabilizes SLC7A11 through deubiquitination [50]. This study reveals a mechanism by which USP5 regulates SLC7A11 independently of the ubiquitin system, expanding the regulatory network of SLC7A11 and further highlighting the heterogeneity of DUB functions in different cancers. Moreover, the positive correlation between USP5 and SLC7A11 provides theoretical support for clinical combination therapies (Erastin and USP5 inhibitors).

The interaction and co-localization of YBX3 and SLC7A11 establish the link between YBX3 and the regulation of ferroptosis. Unlike the mechanism in liver cancer, where circPIAS1 inhibits ferroptosis through the miR-455-3p/NUPR1 axis, YBX3 directly targets SLC7A11 and destabilizes it through lysosomal degradation [24]. In the context of USP5 knockout, silencing YBX3 restores SLC7A11 levels and rescues ferroptosis resistance, suggesting a strict hierarchical regulatory relationship within the USP5/YBX3/SLC7A11 axis.

Patient-derived organoids and xenograft models confirmed that USP5 knockout significantly inhibits tumor growth and enhances ferroptosis sensitivity, with Fer-1 partially reversing this effect. These results resemble the efficacy of ferroptosis therapies targeting LSH in liver cancer and GPX4 inhibitors in glioma, yet the regulatory mechanism of the USP5/YBX3 axis is more specific [19, 50]. Furthermore, the downregulation of SLC7A11 caused by USP5 loss suggests its potential as a biomarker for predicting the efficacy of ferroptosis inducers. Compared to traditional chemotherapy, inhibitors targeting the USP5/YBX3 interaction or the lysosomal pathway may reduce systemic toxicity, providing a new approach for the precision treatment of CRC.

**Fig. 7:**
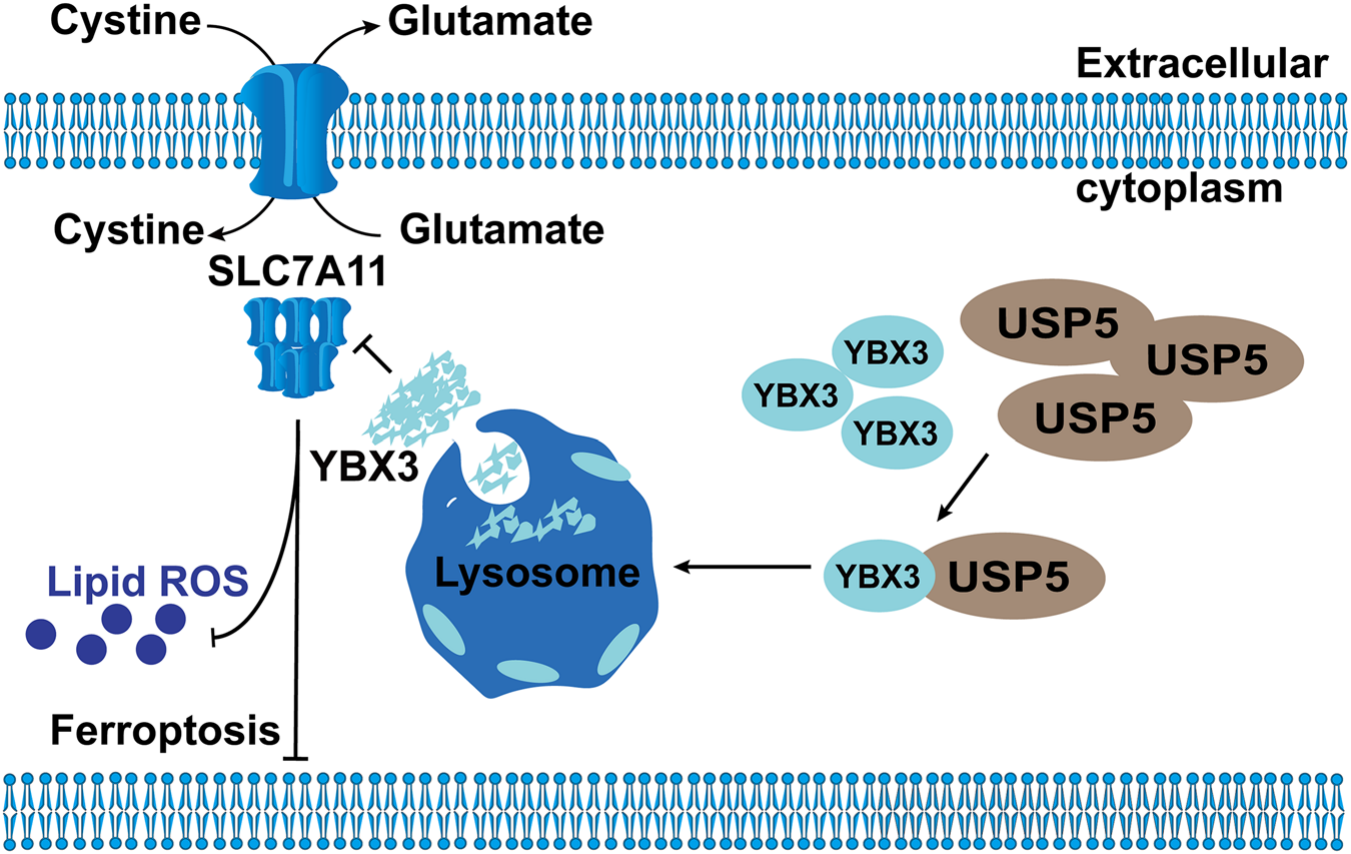
Schematic model of the role of USP5 in regulating the YBX3/SLC7A11 to inhibit ferroptosis. In the presence of USP5, USP5 promotes the translocation and aggregation of YBX3 in the lysosome, thereby mediating the degradation of YBX3 to prevent the degradation of SLC7A11 and inhibit ferroptosis in CRC.

This study elucidates the unique mechanism by which the USP5/YBX3/SLC7A11 axis regulates ferroptosis in CRC, offering a multidimensional analysis: USP5 stabilizes SLC7A11 by degrading YBX3 via the lysosomal pathway, balancing autophagy and ferroptosis (Fig. 7). Compared to other cancers, USP5’s function shifts from relying on ubiquitination to utilizing the lysosomal pathway, and YBX3 transitions from a pro-survival factor to a ferroptosis-sensitive factor. These findings not only expand our understanding of the functional diversity of DUBs but also lay the theoretical foundation for the development of novel therapies targeting the lysosomal-ferroptosis axis. Future research should further explore whether YBX3 is influenced by other molecular chaperones or regulatory factors, and investigate the structural basis of the USP5/YBX3 interaction and its dynamic regulation in the tumor microenvironment to promote clinical translation.

## Materials and Methods

### Primers in PCR

All oligonucleotides, including PCR primers and shRNAs, were chemically synthesized by Tsingke Biotechnology (Beijing, China) using solid-phase phosphoramidite chemistry with HPLC purification (purity > 98%).

**Table:** 

USP5 sgRNA1 Forward:	5′-CACCGTGTCAGTATTACCGACGATC-3′
USP5 sgRNA1 Reverse:	5′-AAACGATCGTCGGTAATACTGACAC-3′
USP5 sgRNA2 Forward:	5′-CACCGTGGGCTTACCGGCGTGTCGA-3′
USP5 sgRNA2 Reverse:	5′-AAACTCGACACGCCGGTAAGCCCAC-3′
ATG5 sgRNA1 Forward:	5′-AAACTCAATCGGAAACTCATGGAAC-3′
ATG5 sgRNA1 Reverse:	5′-CACCGTTCCATGAGTTTCCGATTGA-3′
ATG5 sgRNA2 Forward:	5′-CACCGCCCTTTAGAATATATCAGGT-3′
ATG5 sgRNA2 Reverse:	5′-AAACACCTGATATATTCTAAAGGGC-3′
USP5-Mlu I-Forward:	5′-CGACGCGTATGGCGGAGCTGAGT-3′
USP5-XhoI-Reverse:	5′-CCGCTCGAGGCTGGCCACTCT-3′
USP5-C335A Forward:	5′-CTGGGTAACAGCGCCTACCTCAACTCTGT-3′
USP5-C335A Reverse:	5′-AGAGTTGAGGTAGGCGCTGTTACCCAGGT-3′
USP5 C-ZnF Reverse:	5′-CCGCTCGAGTGCCTGCACCTCCTG-3′
USP5 ZnF Forward:	5′-CGACGCGTATGTGGGATGGGGAAGTA-3′
USP5 ZnF Reverse:	5′-CCGCTCGAGTGTCTTCTGCATCTT-3′
USP5 C Box Forward:	5′-CGACGCGTATGGACAAGACGATGACT-3′
USP5 C Box Reverse:	5′-CCGCTCGAGCGGAGTGACCAGGGG-3′
USP5 U BA1 Forward:	5′-CGACGCGTATGGATGAGCCCAAAGGT-3′
USP5 U BA1 Reverse:	5′-CGACGCGTATGGATGAGCCCAAAGGT-3′
USP5 H Box Forward:	5′-CGACGCGTATGGACATCTCAGAGGGC-3′
YBX3 shRNA1 Forward:	5′-CCGGCGGTTCATCGAAATCCAACTTCTCGAGAAGTTGGATTTCGATGAACCGTTTTT-3′
YBX3 shRNA1 Reverse:	5′-AATTCAAAAACGGTTCATCGAAATCCAACTTCTCGAGAAGTTGGATTTCGATGAACCG-3′
YBX3 shRNA2 Forward:	5′-CCGGCCGTCTGTTCGCCGTGGATATCTCGAGATATCCACGGCGAACAGACGGTTTTTG-3′
YBX3 shRNA2 Reverse:	5′-AATTCAAAAACCGTCTGTTCGCCGTGGATATCTCGAGATATCCACGGCGAACAGACGG-3′

### Plasmids

Plasmids encoding HA-SLC7A11 and HA-YBX3 were purchased from Miaoling Bioscience, while full-length, truncated, and deletion mutants of Flag-USP5, as well as MYC-SLC7A11, ECFP-USP5, EGFP-SLC7A11, ECFP-YBX3, and EGFP-YBX3 were constructed using molecular cloning techniques.

### Chemicals and reagents

Erastin (Selleck, S7242), Ferrostatin-1 (MedChemExpress, S7243), Z-VAD-FMK (TOPSCIENCE, T6013), Necrostatin-1(TOPSCIENCE, 4311-88-0), Cycloheximide (MedChemExpress, HY-12320), CQ (MedChemExpress, HY-17589A), FerroOrange (Cell Signaling Technology 36104S), Lyso Tracker^TM^ Deep Red Probe (ThermoFisher, L12492), MG132 (Selleck, S2619), Protein A/G Beads 4FF (Smart-Lifesciences, SA032025), FreeStyle^TM^ Max (ThermoFisher,16447100).

### Co-immunoprecipitation

Cells were harvested in NP40 lysis buffer containing 150 mM NaCl, 50 mM Tris-HCl pH 8.0, 1% NP40 supplemented with protease inhibitors (10 μg/mL aprotinin, 10 μg/mL leupeptin, and 1 mM phenylmethylsulfonyl fluoride), and the lysates were centrifuged at high speed to remove insoluble debris. Then, the proteins were incubated with the indicated antibodies together with Protein A/G beads (Roche) for overnight at 4 °C. And the beads were washed with IP wash buffer (200 mM NaCl, 50 mM Tris-HCl pH 8.0, 0.1% NP40) for three times and boiled with 1× SDS loading buffer. The interacted proteins were detected using the indicated primary antibodies.

### Cell culture and transfection

The RKO, LoVo, HCT116, NCM460, HCT15, SW48, DLD1, SW480, HT29, and HEK293T cell lines were obtained from the China Center for Type Culture Collection (CCTCC). Cells were maintained under the culture conditions recommended by the supplier, which included appropriate growth media and incubation parameters. Cell lines were authenticated within 6 months of use via 16-locus STR profiling (BGI Genomics) and tested monthly for mycoplasma contamination using MycoAlert™ PLUS (Lonza, LT07-710), with all results confirming absence of contamination. All cells were cultured at 37 °C in Dulbecco’s Modified Eagle Medium (DMEM) with 10% fetal bovine serum (FBS) and 1% antibiotic (penicillin-streptomycin). FreeStyle™ Max DNA in vitro transfection reagent was used for transfection. The FreeStyle™ Max reagent and DNA (1:2 ratio) were mixed and diluted in serum free DMEM for 10 ~ 15 min at room temperature. The FreeStyle™ Max-DNA mixture was then added to the subconfluent cell culture for cell transfection.

### Cell viability and colony formation assay

In the CCK-8 assay for evaluating cell proliferation activity, cells were seeded into 96-well plates at a density of 3000 cells per well and allowed to adhere. After seeding, cells were treated with either Erastin alone or in combination with Fer-1 (5 µM) for 48 hours to assess cell viability. Following treatment, 10 μL of CCK-8 solution (Dojindo Laboratories, Japan) was added to each well, and the plates were incubated at 37 °C with 5% CO₂ for 1 h. Absorbance at 450 nm (ELx800, BioTek, USA) was then measured using a microplate reader to evaluate cell viability. For the colony formation assay, cells were seeded in six-well plates at a density of 500 cells per well and cultured for 14 days or at 250 cells per well for 7 days. Colonies were then stained using crystal violet, followed by imaging for analysis.

### Xenograft tumor growth model

Female BALB/c nude mice (aged 5–6 weeks) were utilized for xenograft studies, with all procedures strictly adhering to the Guide for the Care and Use of Laboratory Animals under approval from the Institutional Animal Care and Use Committee of the University of South China. Mice were randomly assigned to experimental groups via computer-generated randomization (Excel RAND function) performed by an independent researcher to minimize selection bias. To ensure objective assessment, investigators conducting endpoint measurements—including tumor volume monitoring using vernier calipers (initiated on day 5 post-inoculation and performed every 48 h) and final tumor weighing—were blinded to group allocation throughout the study. Similarly, histopathological analyses were conducted by pathologists unaware of treatment conditions. However, treatment administration was not blinded due to visible procedural requirements for drug injection. Single-cell suspensions (5 × 10⁶ cells in 200 μL saline) of HCT116 control, HCT116-sgUSP5, and HCT116-sgUSP5+shYBX3 lines were subcutaneously injected into the dorsal flanks. For pharmacological intervention, mice received intraperitoneal injections of either vehicle control (DMSO diluted in PBS) or 0.5 mg/mL Ferrostatin-1 (formulated from 540 μL stock with 21.6 mL PEG300, 2.7 mL Tween 80, and 29.16 mL saline), with each experimental group containing five mice (*n* = 5). No formal sample size calculation was performed for this animal study. A field-standard sample size (*n* = 5 per group) was adopted based on established models in published literature. Following three weeks of treatment, animals were euthanized for tumor excision and downstream analysis.

### Lipid peroxidation and ROS assay

C11-BODIPY 581/591 (MCE, HY-D1301, 10 µM) was used to detect lipid peroxide levels. Cells were resuspended in PBS and incubated with the above probes at 37 °C for 30 min, followed by analysis using flow cytometry (BeamCyte, FL-1026). Data analysis was performed with FlowJo software.

### Transmission electron microscopy

HCT116 sgCtrl or sgUSP5 cells (1 × 10^6^) were plated in 10 cm dishes. After 72 hours of treatment with DMSO or Erastin, the cells were fixed using 3 mL of 2.5% glutaraldehyde at room temperature for 1 h. The cells were then centrifuged sequentially at 1000 g, 3000 g, 6000 g, and 12,000 g for 5 minutes each and collected. Next, osmium tetroxide staining was performed on ice for 1 h in the dark. Following staining, cells were washed with uranyl acetate and incubated overnight at room temperature in darkness. After rinsing with ddH2O, ethanol gradient dehydration was applied. The dehydrated samples were then incubated in propylene oxide and resin mixtures at 1:1 and 1:2 ratios, followed by immersion in 100% resin for 4 h. The samples were subsequently placed in plastic molds and allowed to cure at 37 °C overnight. At last, the samples were placed in a 65 °C oven for 48 h. Ultrathin sections were prepared, and the samples were subsequently examined using electron microscopy.

### Reverse transcription and quantitative real-time PCR (qPCR)

Total RNA was extracted from cells using Trizol reagent (Invitrogen, #15596-018CN) following the manufacturer’s instructions. RNA was then reverse transcribed into cDNA using the 1st Strand cDNA Synthesis Kit with gDNA Wiper (Vazyme, #R312-01). mRNA expression levels of target genes were quantified by quantitative PCR (qPCR) using SYBR Green qPCR Mix (Monad, #MQ10101S) on a real-time fluorescence PCR analyzer (Life Real, #QuantReady K9600). The following primer sequences were used for qPCR:

**Table:** 

*SLC7A11* Forward:	5′-TGTGTGGGGTCCTGTCACTA-3′
*SLC7A11* Reverse:	5′-GCAGGGCGTATTATGAGGAG-3′
*β-Actin* Forward:	5′-CATCCGCAAAGACCTGTACG-3′
*β-Actin* Reverse:	5′-CCTGCTTGCTGATCCACATC-3′

### Immunofluorescence

Following 48-hour co-transfection with ECFP-USP5 and EGFP-YBX3 plasmids in HCT116 cells or EGFP-SLC7A11 and ECFP-YBX3 plasmids in HEK-293T cells, live-cell imaging was initiated by replacing culture medium with pre-warmed medium containing 70 nM Lyso Tracker^TM^ Deep Red, followed by 37 °C incubation in the dark for 30 minutes (Note: duration reduced to minimize phototoxicity). After three gentle washes with 37 °C PBS (30 sec/wash), cells were immediately transferred to a live-cell chamber for synchronized three-channel confocal imaging (Zeiss LSM980): ECFP (433/475 nm), EGFP (488/509 nm), and Lyso Tracker^TM^ Deep Red (647/668 nm), with all imaging completed within 20 minutes post-staining to preserve viability. Subsequent 3D reconstruction and Manders’ coefficient-based colocalization analysis were performed using ZEN software.

### IP mass spectrometry experiment

The IP-MS procedure was performed as follows: Cell lysates were prepared using lysis buffer supplemented with protease inhibitors, followed by sonication to enhance cell disruption. Protein concentration was quantified using either BCA or Bradford assay to ensure sufficient USP5 protein amounts. For proteolytic digestion, quantified USP5 samples were incubated with digestion buffer and trypsin at 37 °C for 4 h to overnight, with the reaction terminated by adding equal volume of acidic solution (e.g., TFA). Sample purification was conducted using C18 solid-phase extraction columns according to the manufacturer’s protocol, including washing and elution steps, with subsequent solvent removal using nitrogen drying or lyophilization to obtain dried peptide samples. For MS analysis, dried peptides were reconstituted in MS-compatible solvent (0.1% FA in water/acetonitrile) and loaded via syringe injection. Mass spectrometer parameters (voltage, gas flow, temperature) were optimized for sample characteristics before data acquisition. Finally, raw data were processed using specialized software (MaxQuant/Mascot/Proteome Discoverer) for peptide identification, USP5 quantification, and subsequent bioinformatic analysis.

### Ferrous iron detection

FerroOrange (Dojindo, #F374) was used to assess intracellular Fe^2+^ levels. Cells were cultured in a 96-well plate. After drug treatment, the supernatant was discarded. A working solution of FerroOrange at a concentration of 1 μmol/L was added, and the cells were incubated for 15 min in a 37 °C incubator. Images were captured using the automated cell imaging system (Molecular Devices, ImageXpress Pico, CMOS).

### Patient-derived organoids

Colorectal tumor tissues were obtained postoperatively and processed in the laboratory. The specimens were initially washed with PBS and then mechanically dissociated into small fragments. These fragments were subsequently digested using Collagenase IV at 37 °C for 1 h. The resulting cell suspension was passed through a 40 μm cell strainer to isolate single cells. The isolated cells were resuspended in Matrigel and seeded into 48-well plates. Organoids derived from patient colorectal tumors were cultured in Advanced DMEM/F12 medium (Gibco) supplemented with Primocin (InvivoGen), GlutaMax (Gibco), HEPES (Gibco), B27 (Gibco), N2 supplement (Gibco), SB202190 (MedChemExpress), Y27632 (MedChemExpress), Blebbistatin (MedChemExpress), CHIR99021 (MedChemExpress), A83-01 (Tocris Bioscience), and recombinant human R-Spondin-1 (rhR-Spondin-1; R&D Systems). The cultures were maintained at 37 °C in a 5% CO_2_ atmosphere.

### Calcein/PI Viability Assay for CRC Organoids

CRC organoids were seeded in 96-well plates and treated with DMSO, Erastin, or Erastin + Fer-1 for 48 hours prior to analysis. Following treatment, a working solution of Calcein AM/Propidium Iodide (PI) was prepared (Beyotime, #C2015M). After discarding the supernatant, 100 μL of the working solution was added to each well, and the plate was incubated at 37 °C in the dark for 30 minutes. After incubation, fluorescence was observed using a high-content cell imager (Molecular Devices, ImageXpress Pico, CMOS) (Calcein AM: green fluorescence, Ex/Em = 494/517 nm; PI: red fluorescence, Ex/Em = 535/617 nm).

### PI staining flow cytometry cell death analysis

In order to assess cell viability, this experiment uses propidium iodide (PI) staining combined with flow cytometry for cell death analysis. First, an appropriate cell line is selected and cultured to the logarithmic growth phase to ensure optimal cell condition. Before the experiment, cells are washed with PBS (phosphate-buffered saline) to remove impurities from the culture medium. For adherent cells, gentle trypsinization is performed to remove non-adherent cells. After treatment, cells are collected and centrifuged to remove the culture medium, then resuspended in PBS to an appropriate concentration, typically 1 × 10⁶ cells/ml. Next, PI dye is added to the cell suspension at a concentration of 50 μg/ml, and cells are incubated at room temperature, protected from light, for 20–30 min. During this process, PI can only enter dead cells with damaged membranes, and therefore, live cells do not stain with PI. After staining, cells are analyzed using flow cytometry. The flow cytometer is set to excite at 488 nm, with a red fluorescence channel ( > 600 nm) selected to detect PI fluorescence signals. At the end of the experiment, the flow cytometer software outputs cell distribution data, typically performing two-parameter analysis (such as FSC and PI staining intensity).

### ATP viability assay for CRC organoids

CRC organoids were seeded in 96-well plates and treated with either DMSO or Erastin for 72 h. After treatment, cell viability was assessed using the Organoid Viability ATP Assay Kit (BioGenous, #E238003). The plate was removed from the incubator and equilibrated to room temperature for 10 min. An equal volume of detection reagent was added to each well (1:1 ratio). The plate was subjected to linear shaking at 1000 rpm for 5 min, followed by incubation at room temperature for 20 min. Chemiluminescence was measured at 560 nm using a microplate reader.

### Malondialdehyde (MDA) testing

In this experiment, the MDA levels in tumor samples from the DMSO and Fer-1 groups were measured using the Lipid Peroxidation (MDA) Assay Kit (Beyotime, S0131S). First, TBA was dissolved in the TBA solution to prepare a 0.37% TBA stock solution. If necessary, the solution can be heated to 70 °C to aid dissolution. Next, the MDA detection working solution was prepared and freshly made according to the experimental requirements. Then, the samples were homogenized or lysed using a lysis buffer, followed by protein concentration measurement using the BCA Protein Assay Kit (Epizyme Biotech, ZJ101). Afterward, samples and standards were added to test tubes, and 0.2 mL of the MDA detection working solution was added. The mixture was heated at 100 °C for 15 minutes in a water bath, then cooled to room temperature and centrifuged at 1000 g for 10 minutes. The supernatant was then used for absorbance measurement. Absorbance was measured using a microplate reader at 532 nm. If this wavelength is not convenient, measurements can also be taken between 530 and 540 nm. The data was calculated by comparing the absorbance of the samples to the standard curve to determine the MDA concentration in the samples.

### Protein extraction and western blot analysis

Cells were dissociated by digestion with 0.25% trypsin (containing 0.02% EDTA) at 37 °C for 1.5 minutes. After microscopic confirmation that the cells had fully detached from the six-well plates, digestion was stopped by adding two volumes of complete growth medium. The cell suspension was transferred to a 1.5 mL centrifuge tube and centrifuged at 500 × g for 5 minutes at 4 °C. Supernatants were carefully aspirated to avoid cell loss, and the pellets were washed once with pre-chilled PBS followed by a repeat centrifugation under the same conditions. Cell pellets were resuspended in 100 µL freshly prepared 1× SDS lysis buffer (50 mM Tris-HCl pH 8.0, 1% SDS, 5% β-mercaptoethanol), vortexed for 30 seconds to ensure complete resuspension, and immediately heated in a metal bath at 95 °C for 15 minutes (with tubes fully immersed). After heat denaturation, samples were chilled on ice for 2 minutes and centrifuged at 12,000 × g for 10 minutes to collect the supernatant. Lysates were aliquoted and stored at −80 °C for batch processing. Protein concentrations were determined using the BCA assay, and equal amounts of protein were separated on 10% SDS-PAGE gels (80 V for stacking, 120 V for resolving). Proteins were transferred to PVDF membranes by wet transfer at constant 100 V for 60 min. Membranes were blocked in TBST containing 5% nonfat milk on a shaker at room temperature for 1.5 h, then incubated overnight at 4 °C with primary antibodies diluted in blocking buffer (e.g., Anti-XX, 1:1000). Following three stringent washes in TBST (10 minutes each), membranes were incubated with HRP-conjugated secondary antibodies (1:5000) on a shaker at room temperature for 1 h. Signals were developed using ECL chemiluminescence reagents (Bio-Rad) and imaged with the ChemiDoc system. Each experimental group included three independent biological replicates. The primary antibodies used for western blot analysis are as follows: anti-USP5 (Proteintech, 10473-1-AP;1;3000), anti-YBX3 (Bethyl, A303-070A-T;1:2000), anti-SLC7A11 (Cell Signaling Technology, 98051;1:3000), anti-β-tubulin (Cell Signaling Technology, 2146;1:5000), anti-Flag (Smart-Lifesciences, SLAB0102;1: 5000), anti-HA (Smart-Lifesciences, SLAB0202;1:5000), anti-Myc (Abclonal, AE010;1:5000), anti-ATG5 (Cell Signaling Technology, 12994;1:3000).

### Statistical analysis

All statistical analyses were performed using GraphPad Prism 8.0 and ImageJ software. Prior to hypothesis testing, data distribution normality was confirmed by Shapiro-Wilk test (α = 0.05), and variance homogeneity between compared groups was verified using Brown-Forsythe test. Based on these validated assumptions, appropriate parametric tests were applied: unpaired Student’s t-tests for comparisons between two groups, and one-way or two-way ANOVA followed by Tukey’s multiple comparisons test for multi-group analyses. All data are presented as mean ± standard deviation (SD), with individual sample sizes (representing patients, mice, or independent biological replicates) explicitly depicted as data points in figures and detailed in corresponding legends. A minimum of three biological replicates was included in all experiments. Statistical significance thresholds were set at *P < 0.05, **P < 0.01, and ***P < 0.001.

## Supplementary information

Supplemental Figures

Western blot data

## Data Availability

The analysis of public datasets including The Cancer Genome Atlas (TCGA) was conducted using data available from the Genomic Data Commons Portal (https://portal.gdc.cancer.gov/). All other data supporting the findings of this study are available within the article and its Supplementary Information files, or from the corresponding author upon reasonable request. Source data are provided with this paper.
