# Supplementary material for: USP5 regulates ferroptosis in colorectal cancer by targeting the YBX3/SLC7A11 axis through lysosomal degradation
Source: Cell Death Dis. 2025 Nov 10;16(1):822. doi: 10.1038/s41419-025-08146-2 (PMC12603231; doi:10.1038/s41419-025-08146-2)
Supplement: Supplementary file 1 — Supplemental Figures [file 41419_2025_8146_MOESM1_ESM.docx]

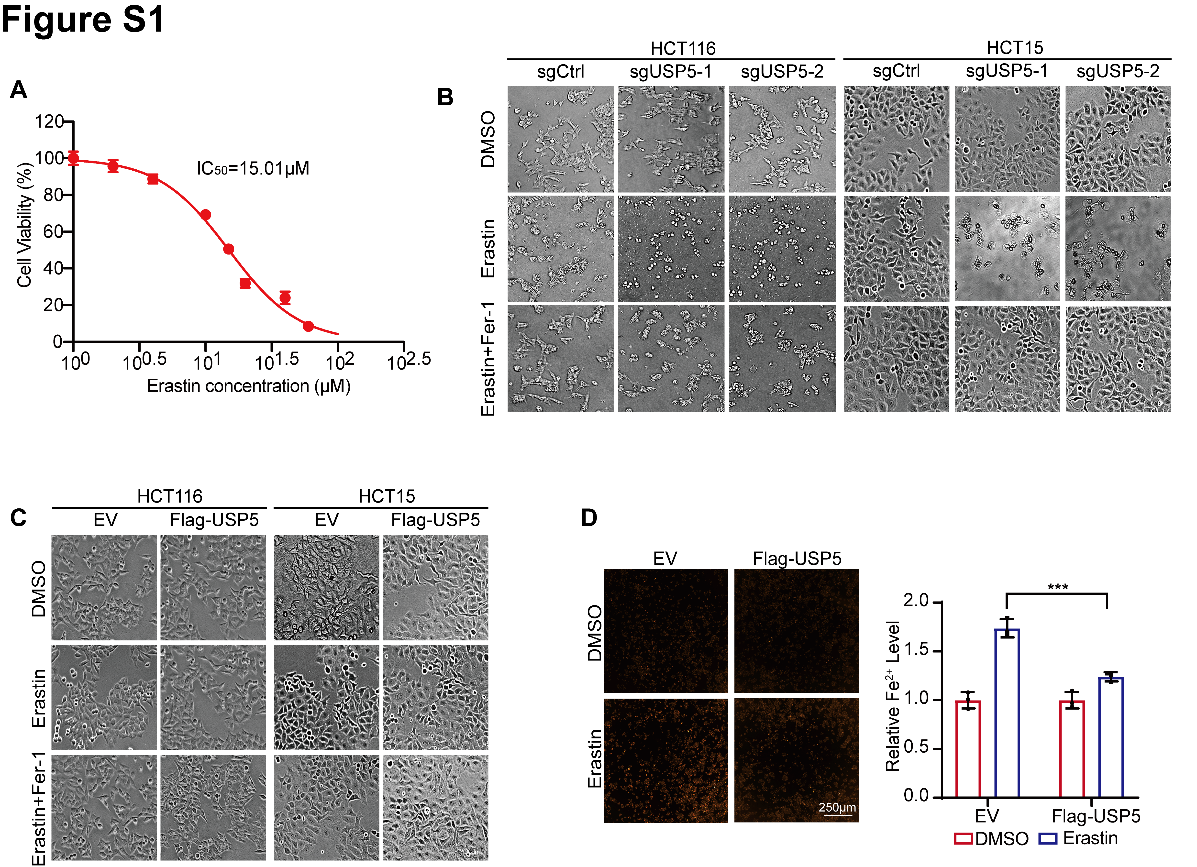


**Figure S1 USP5 knockout sensitizes CRC cells to ferroptosis**

(**A)** Determination of IC_50_ in normal human colorectal cancer cells. (**B**) In HCT116 and HCT15 cell lines, USP5 knockout cells were treated with erastin (15 µM) for 72 hours. Cell viability was subsequently assessed using the Capture images under the microscope. (**C**) In HCT116 and HCT15 cell lines, USP5 overexpressing cells were treated with erastin (20 µM) for 72 hours. Cell viability was subsequently assessed using the Capture images under the microscope. (**D**) Intracellular Fe^2+^ levels were determined using ferroOrnge in USP5 overexpression cells with quantitative analysis presented. Data are shown as means ± SDs. **p* < 0.05, ***p* < 0.01, ****p* < 0.001, one-way ANOVA.

**
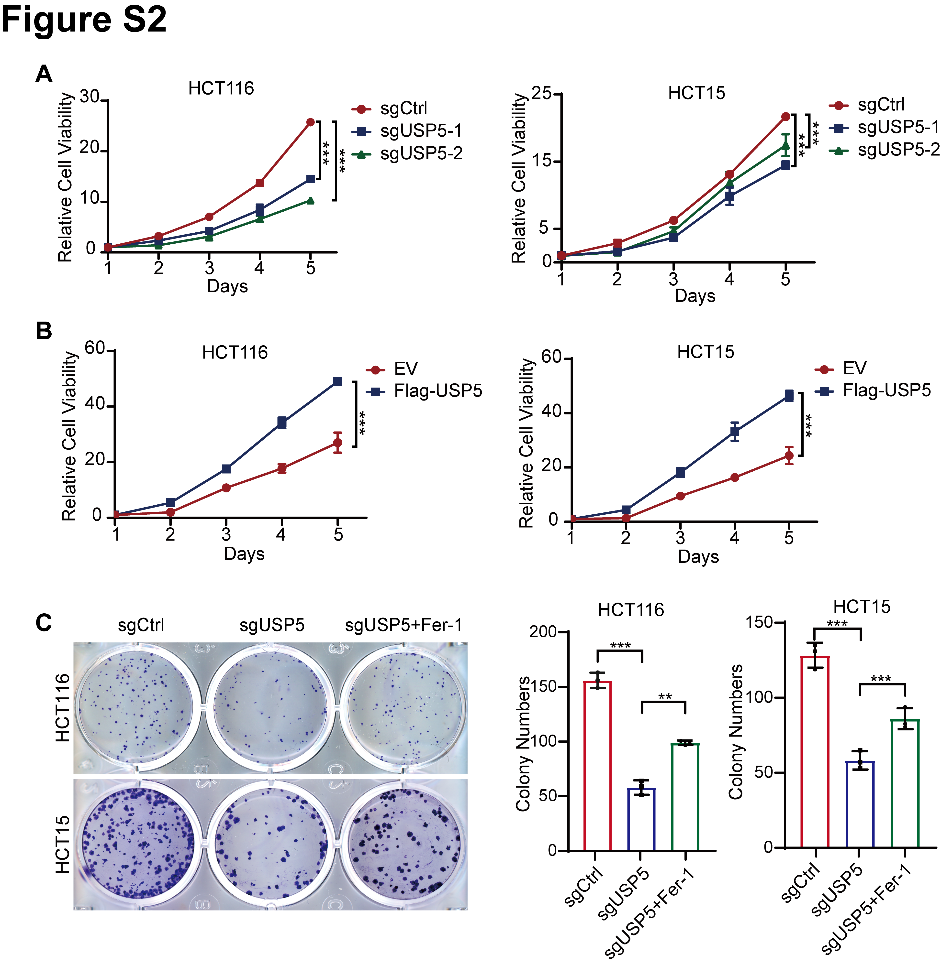
**

**Figure S2 USP5 Drives Colorectal Cancer Progression through Enhanced Proliferation, Migration**

**(A)** Cell viability assay was performed over a 5-day period in HCT116 and HCT15 cells with control sgRNA (sgCtrl) and two different USP5 knockout groups (sgUSP5-1 and sgUSP5-2) (n=6). **(B)** Cell viability assay was performed over a 5-day period in HCT116 and HCT15 cells with either control vector (EV) or USP5 overexpression (Flag-USP5) (n=6). **(C)** Colony formation assay was conducted in HCT116 and HCT15 cells with control sgRNA (sgCtrl) and USP5 knockout (sgUSP5) following Erastin treatment, with or without additional fer-1(5 µM) treatment. Representative images of crystal violet-stained colonies are shown on the left, and quantification of colony numbers is presented on the right (n=3). Data are shown as means ± SDs. **p* < 0.05, ***p* < 0.01, ****p* < 0.001, one-way ANOVA and two-way ANOVA.


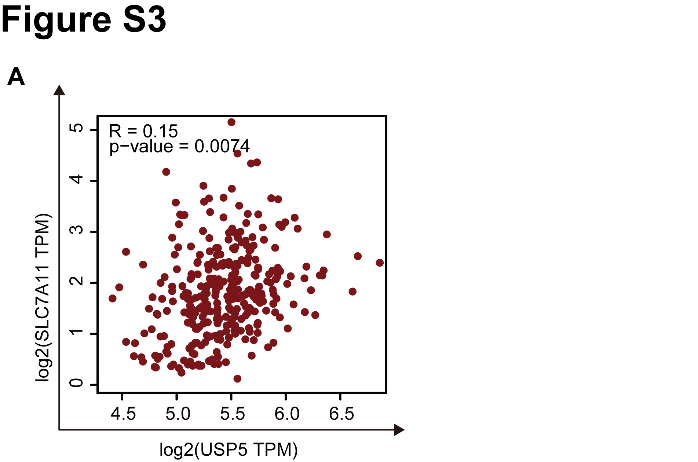


**Figure S3 USP5 regulates ferroptosis by stabilizing SLC7A11**

**(A)** Analysis of the correlation between USP5 and SLC7A11 using TCGA database.


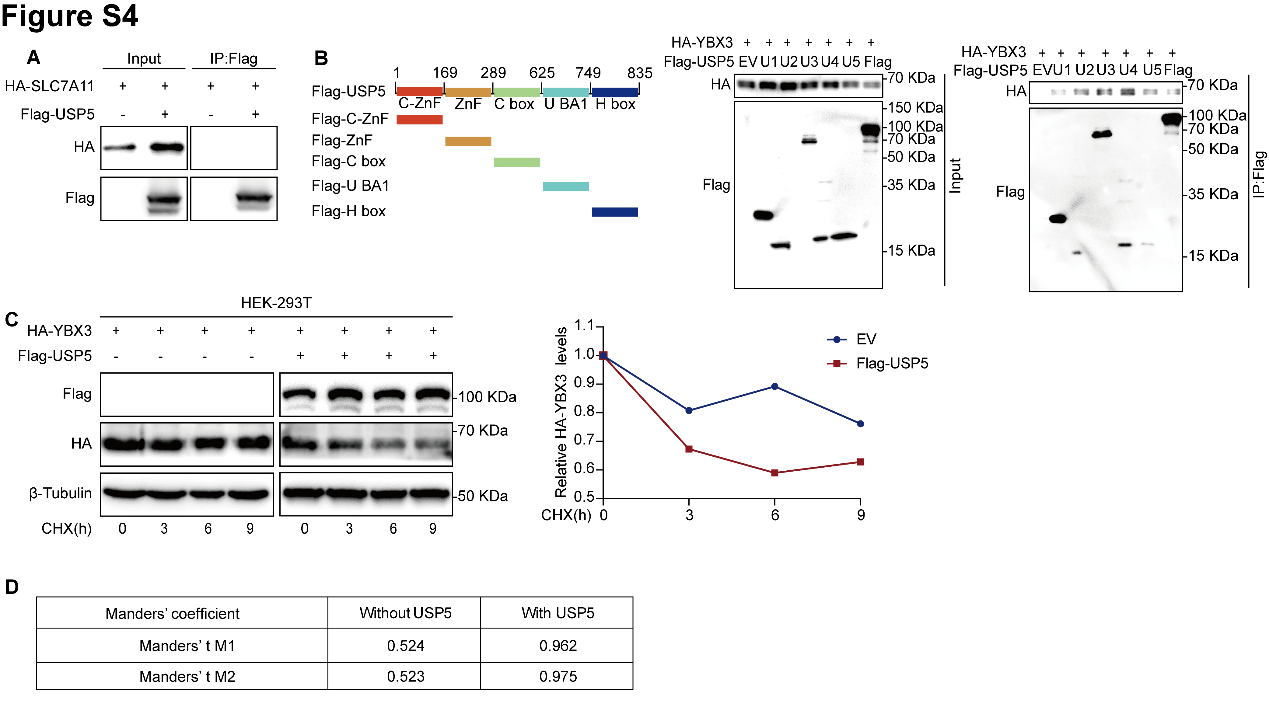


**Figure S4 USP5 promotes lysosomal degradation of YBX3**

**(A)** Perform transient transfection of Flag-USP5 and HA-SLC7A11 vector into 293T cells and use exogenous Co-IP to detect interactions. **(B)** Co-transfect full-length Flag-tagged USP5 and its five truncated mutants with HA-YBX3 into HEK293T cells for 48 hours, then prepare cell lysates and perform IP using specific antibodies. **(C)** Western blotting analysis exhibiting YBX3 remaining level at indicated time in HCT16 with USP5 knockout and treatment with CHX (100 μg/mL). **(D)** Quantification of Manders’ colocalization coefficients (tM1 and tM2) for YBX3 and Lysosome in cells without or with USP5 overexpression. Data are shown as means ± SDs. **p* < 0.05, ***p* < 0.01, ****p* < 0.001, two-way ANOVA.


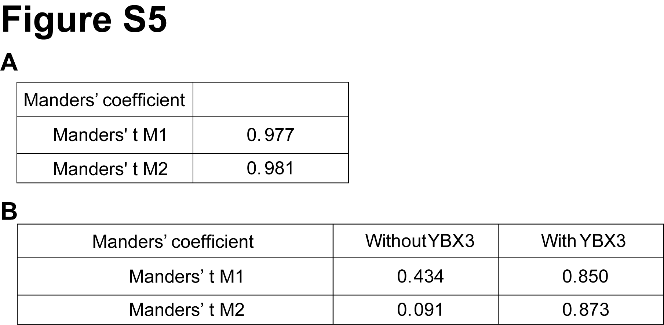


**Figure S5 YBX3 regulates cellular ferroptosis by lysosomal degradation of SLC7A11**

**(A)** Quantification of Manders’ colocalization coefficients (t M1 and t M2) for YBX3 and SLC7A11 in cells. **(B)** Quantification of Manders’ colocalization coefficients (t M1 and t M2) for SLC7A11 and Lysosome in cells without or with YBX3 overexpression.
